# Pan-esophageal squamous papillomatosis with high grade dysplasia managed with endoscopic submucosal dissection

**DOI:** 10.1055/a-2690-1913

**Published:** 2025-09-11

**Authors:** Yong Sul Kim, Sergei Vosko, Sunil Gupta, Brian Lam, Clarence Kerrison, Nicholas Graeme Burgess, Michael J. Bourke

**Affiliations:** 1 8539 Department of Gastroenterology and Hepatology, Westmead Hospital, Sydney, Australia; 2 216997 Department of Medicine, The University of Sydney Westmead Clinical School, Sydney, Australia; 3 216997 Department of Gastroenterology and Hepatology, The University of Sydney Westmead Clinical School, Sydney, Australia

A 63-year-old woman presented to her local gastroenterologist with a two-year history of dysphagia. A barium swallow demonstrated free passage of liquid barium and a barium-soaked marshmallow but noted a small irregularity of the mucosal surface in the mid esophagus ( Fig. 1 ). Subsequent gastroscopy revealed circumferential diffuse white-pink plaques with a cobblestone appearance, extending 23–38 cm from the incisors. Biopsies revealed squamous papillomatosis with multifocal low and high grade dysplasia. Computed tomography of the neck, chest, and abdomen was unremarkable. She was referred for consideration of endoscopic submucosal dissection (ESD).

**Fig. 1:**
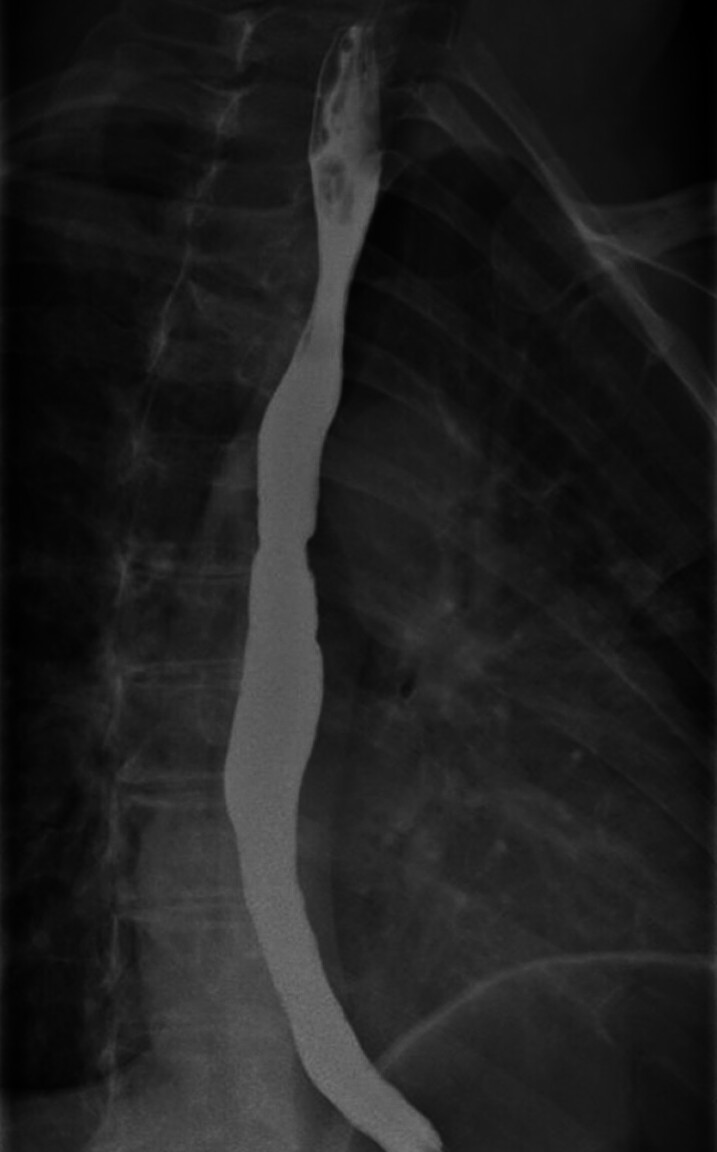
Barium swallow demonstrating free passage of liquid barium, with slightly irregular wall in the mid esophagus.

The lesion was assessed under high-definition white light and narrow-band imaging to determine the boundaries of the lesion ( Fig. 2 ). This was marked with a 1.5-mm DualKnife J (Olympus, Tokyo, Japan). Following a submucosal lift (Gelofusine, indigo carmine, diluted adrenaline) and proximal mucosal incision, a ¾ circumferential submucosal tunnel was created. Once completed, the distal margin was incised to complete the tunnel. Clip-and-snare traction was utilized to facilitate further submucosal dissection, en bloc resection, and retrieval ( Video 1 ). There were no adverse events. Final histological examination revealed extensive squamous intraepithelial neoplasia with mixed low and high grade dysplasia, and multifocal changes of squamous papillomatosis featuring hyperplastic squamous epithelium overlying finger-like cores of lamina propria. There was no evidence of recurrence at 72 months. As expected, a post-ESD stricture developed and was managed with serial dilations over six years ( Video 1 ).

**Fig. 2:**
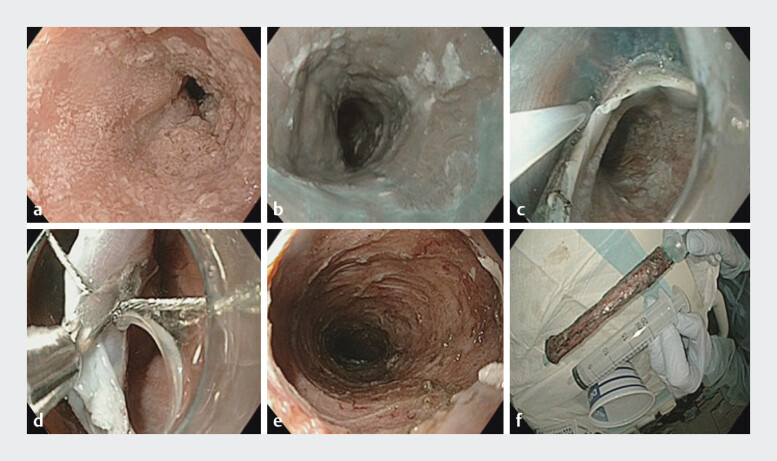
Endoscopic images showing diffuse esophageal papillomatosis in the mid-distal esophagus. **a** In high definition white light. **b** In narrow-band imaging. **c** Mucosal incision and submucosal dissection. **d** Clip-and-snare method to create traction. **e** Large mucosal defect. **f** The resected specimen in white light placed over a nasopharyngeal tube.

En bloc resection of pan-esophageal squamous papillomatosis with multifocal low and high grade dysplasia via endoscopic submucosal dissection.Video 1

Esophageal papillomatosis may be discovered incidentally as a solitary papular lesion. It is generally a benign growth and usually asymptomatic 1 . Diffuse esophageal involvement is exceptionally rare but can carry a risk of malignant transformation. Although the etiology is unclear, the human papillomavirus infection and chronic mucosal irritation have been proposed as potential causes 2 3 . As demonstrated, ESD is a safe and effective treatment modality that facilitates organ-preserving eradication, which is sustained long-term.

Endoscopy_UCTN_Code_TTT_1AO_2AC

## Notes

Endoscopy E-Videos https://eref.thieme.de/e-videos
*E-Videos* is an open access online section of the journal *Endoscopy* , reporting on interesting cases and new techniques in gastroenterological endoscopy. All papers include a high-quality video and are published with a Creative Commons CC-BY license. Endoscopy E-Videos qualify for HINARI discounts and waivers and eligibility is automatically checked during the submission process. We grant 100% waivers to articles whose corresponding authors are based in Group A countries and 50% waivers to those who are based in Group B countries as classified by Research4Life (see: https://www.research4life.org/access/eligibility/ ). This section has its own submission website at https://mc.manuscriptcentral.com/e-videos .
